# Integrating climate-smart rice agriculture into secondary-level curriculum: lessons from three high schools in the Philippines

**DOI:** 10.1186/s40064-016-3238-6

**Published:** 2016-09-17

**Authors:** Jaime A. Manalo, Katherine P. Balmeo, Jayson C. Berto, Fredierick M. Saludez, Jennifer D. Villaflor, Argie M. Pagdanganan

**Affiliations:** Development Communication Division, Philippine Rice Research Institute, 3119 Maligaya, Science City of Muñoz, Nueva Ecija Philippines

**Keywords:** Climate-smart rice agriculture, Infomediaries, Teaching climate change, Climate change

## Abstract

Climate change (CC) is an urgent and highly relevant topic that must be integrated into the school curriculum. Literature on CC integration, however, is scarce, let alone literature on integrating climate-smart rice agriculture (CSRA). Bringing CSRA lessons into the classroom means the chance is higher that climate-smart technologies on rice will reach even the most far-flung areas of the Philippines, which stand to be among the most vulnerable as regards the negative impacts of CC. This paper shares experiences drawn from three high schools in the Philippines on integrating CSRA into their curriculum. The research centers on appropriate teaching tools/strategies, push and exogenous factors in CSRA integration, and the types of information that are likely to be shared by the students with their farmer-parents or other farmers in their communities. Surveys among participating students (n = 155) and three focus group discussions among key school officials were conducted. Different teaching methods and/or tools were found to be generally useful in various contexts. Photos and videos, however, emerged as the most effective tools across sites. The livelihood source of the students does have a bearing on the complexity of messages that they can convey. Students from rice-farming households can competently discuss even highly complex adaptation and mitigation information with their farmer-parents or other farmers. Thorough message-framing is necessary to maximize student involvement as well as to increase production of education–entertainment (edutainment) materials to be utilized in teaching. This study, in general, contributes to CC education by bringing in best-fit practices in teaching tools and strategies to mobilize students to act on urgent matters relating to the impacts of CC. It also advises on considering exogenous factors that might affect CC education by taking into account those that are equally capable of shaping students’ perception and knowledge.

## Background

Climate change (CC) is an urgent and highly relevant topic that must be integrated into the school curriculum. In the Philippines, for instance, an annual average of 20 typhoons enter its area of responsibility (PAG-ASA 2011), a few of which wreak havoc on lives and livelihoods, particularly in agriculture. Addressing issues relating to CC requires concerted efforts, which can be done only through proper education of all key stakeholders. Scholars have noted that knowledge is a significant motivator of behavioral change (Harker-Schuch and Bugge-Henriksen 2013).

As we write, it seems that efforts to integrate CC lessons into the curriculum at the global level remain fragmented, and at best being highly debated. Reasons for fragmentation and debate are beliefs of teachers, inadequacy of time, inflexible curricula, methodological dilemmas, and funding deficiencies (Duschl 1990; Waters-Adams 2006; Ratinen et al. 2013; White et al. 2014; Herman et al. 2015).

It has been observed that teachers have the tendency to align their teaching according to their own beliefs (Duschl 1990; Waters-Adams 2006), and in many instances, owing to the absence of a fully designed curriculum, they rely on doing their own research by searching for books and references that strengthen their views on a given subject (Wigfield and Eccles 2000; Disinger 2001; Choi et al. 2010). Confusion in key CC terminologies has also been noted (greenhouse gas emissions and global warming, for instance) (Ratinen et al. 2013; Herman et al. 2015). These failings can lead to misinformation.

If teaching CC topics in general is not adequately covered in the literature, CC as it relates to agriculture is all the more left out. Most of the materials available dwell on general CC concepts (Ratinen et al. 2013; Herman et al. 2015; Quigley 2015) with strong focus on mitigation efforts and not much on adaptation strategies. Furthermore, most of the literature available on the subject is from the United States and other developed countries, not much from Asia (Lambert and Bleicher 2013; Herman et al. 2015).

This paper seeks to fill in the gaps by discussing an initiative to integrate climate-smart rice agriculture (CSRA) lessons in the high school curriculum in the Philippines. Climate-smart agriculture is “an approach developing the technical, policy and investment conditions to achieve sustainable agricultural development for food security under climate change” (Palombi and Sessa 2013, p. ix). It has three pillars: (1) sustainably increasing agricultural productivity and incomes; (2) adapting and building resilience to CC; and (3) reducing and/or removing greenhouse gas emissions, where possible (Palombi and Sessa 2013, p. ix). Focusing on rice agriculture is extremely relevant to the Philippines, especially since many of the negative repercussions of CC are being felt in rural rice-farming communities.

This study was conducted under the auspices of the Infomediary Campaign (www.infomediary4d.com) of the Philippine Rice Research Institute (PhilRice). Essentially, the Campaign engages young people, 13–16 years old, in agriculture by mobilizing them as CSRA infomediaries or information providers. To be an infomediary, students must be able to either read, surf, or text information on rice and share it with their farmer-parents or other farmers in their communities. In the Read Component, publications on rice, which the students can borrow, are given to participating schools. In the Surf Component, the students are introduced to PinoyRice, which is a website that contains plenty of information on rice production in the Philippines. The Text Component introduces the students to the PhilRice Text Center (PTC), which is a short messaging service (SMS) facility that responds to all queries on rice. All students participating in the Campaign are registered for this facility. All SMSs they send go to a separate folder for content analysis. The PTC and PinoyRice were both developed by the then Open Academy for Philippine Agriculture.

Nationwide, 108 schools participated in the Campaign; all were encouraged to integrate CSRA into their school curriculum in the June 2014 to March 2015 academic year. The Campaign is in collaboration with the Philippine Department of Education and the Consultative Group on International Agricultural Research Program on Climate Change, Agriculture, and Food Security (CGIAR CCAFS). Prior to integration, the participating teachers were trained on CSRA at the Central Experiment Station of PhilRice, the country’s lead agency for rice research and development. Integration meant that the teachers should teach CSRA in their respective schools. Participating teachers were provided with three CSRA modules and a teaching guide on *Climate Change 101, Climate Change Mitigation Strategies*, and *Climate Change Adaptation Strategies*. All three modules are in Filipino.

*Climate Change 101* contains basic information on the science of CC and its impacts on rice production that help avoid communicating fear among students. It is known that fear does not motivate action when it comes to CC adaptation (IPCC 2007). The *Climate Change Mitigation Strategies* module tackles the different ways by which human interventions either aggravate or alleviate the CC phenomenon. The *PalayCheck* System, which is an integrated crop management system and PhilRice’s banner program for favorable ecosystems, is extensively discussed in this module. *PalayCheck* is composed of a set standards that, if achieved, will help farmers attain higher yields. The *Climate Change Adaptation Strategies* module contains strategies for rice farmers to adapt to the impacts of CC. *Palayamanan* or the rice-based farming system, which is PhilRice’s banner program for unfavorable ecosystems, is extensively discussed in this module. *Palayamanan* emphasizes crop diversification to lessen the rice-farming household’s reliance on rice alone. Growing high-value crops and raising livestock are taught in this program.

The teaching guide serves as a script for the teachers. This is a necessary intervention to assist teachers in more effectively teaching this subject matter, especially since materials on CC and rice production are not always adequate.

The overarching goal of this Campaign is to strategize on how to properly integrate CSRA lessons in the curriculum of public high schools in the Philippines. In integrating CSRA into the curriculum, the teachers and students are the key actors. The teachers use the appropriate methods to effectively convey CSRA lessons. They also have to pay close attention to the push factors, which can either be internal (high interest, innovativeness) or external (policies and overall teaching environment) to them.

The students, on the other hand, process the CSRA lessons. Some or all of the lessons are shared with farmers depending on the capacity and willingness of the students. Proper integration boosts the chances that CSRA technologies will be disseminated to farmers in rural areas, who are most vulnerable to the ill effects of CC. Specifically, this paper has four objectives: (1) identify the various teaching tools/strategies to be used; (2) present the push factors in CSRA integration; (3) present the exogenous factors that may affect CSRA integration; and (4) identify the types of information that are likely to be shared by the students.

## Methodology

### Sites

Of the 108 sites of the Campaign, we chose to do this research in three schools: Malalag National High School (MNHS) in Sarangani, Libon Agro-industrial High School (LAIHS) in Albay, and Cateel National Agricultural High School (CNAHS) in Davao Oriental. The schools were purposively chosen following three major criteria: success in implementation, evidence of CSRA integration, and location (must be in a rice-farming community).

### Research respondents

The respondents were all students actively engaged in the three schools (MNHS—49; LAIHS—46; and CNAHS—60). The schools had 1000 students each, on average. We had requested the schools to nominate a class to be actively engaged in the Campaign and where crop production would be extensively tackled. Clustered according to their fields of specialization, 36 % of the respondents were crop production majors, while 64 % were noncrop production majors. Some 26 % of the respondents were males, more than half (55 %) of whom were directly involved in farming. Parents of 31 % of respondents were landowners. Key school officials and the teachers trained under this initiative were likewise interviewed as key informants.

### Methods

We used qualitative (focus group discussions [FGDs]) and quantitative (surveys) research methods. An FGD was conducted in each school, participated in by 3–5 people—the teacher trained in the Infomediary Campaign, the school head, the technical-vocational education head, and some teachers who had some involvement in implementing the Campaign. The questions revolved around their efforts to integrate CSRA into their curriculum. On average, each FGD lasted for an hour. Surveys were conducted from November 2014 until February 2015. Questions were evaluative in nature: Were the modules given used by the participating teacher? How effective were the modules in transmitting information? and What was the information taught by the teachers and eventually passed on by the students to the farmers in their communities?

### Analysis

The qualitatively derived data were analyzed thematically. The summary of findings is presented in the tables. Quantitatively derived data were analyzed using descriptive statistics and are presented in graphs.

### Ethical consent

This research is under PhilRice’s Infomediary Campaign being implemented with the Philippine Department of Education and CGIAR CCAFS. Oral consent was secured from the participating teachers. A briefing about the research was also done among students and teachers. For anonymity, the students are not named in this research.

### Conceptual framework

**Fig. 1:**
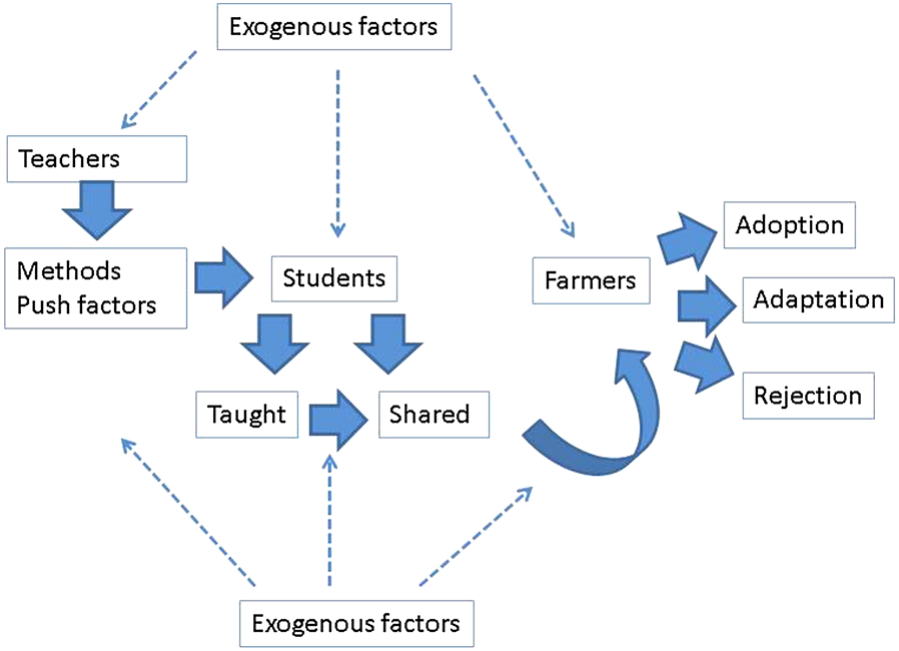
Conceptual framework

This study is situated in the place-based education pedagogy (see Fig. 1). The key principle is to strengthen the connection of the students to the community in which they live (Tytler et al. 2010). “It is intergenerational, multidisciplinary and experiential, uses knowledge and skills in real life situations, is authentically connected to student life worlds, and builds sense of ecological relationship” (Barraza and Bodenhorn 2012, pp. 118–119, drawn from studies of Edwards 2003 and Smith 2002).

CC education and adaptation literature especially on issues surrounding teaching also guide this research. Key gaps observed are issues relating to how to successfully integrate CC in the curriculum (Duschl 1990; Waters-Adams 2006; Ratinen et al. 2013; White et al. 2014; Herman et al. 2015), and hence offer a fertile ground for research. Additionally, issues surrounding how to push young people to serve as allies in addressing the ill effects of CC are left unaddressed in the literature (Harker-Schuch and Bugge-Henriksen 2013).

## Findings and discussion

This section is divided into two major parts consistent with the two frameworks in which this study wishes to contribute. The first major part, which talks about the teaching tools and/or strategies, push factors in teaching CSRA, and exogenous factors that may affect CSRA integration, deals with how this study advances CC education and adaptation literature.

The second major part, which expounds on the topics that were taught to the students and those that were eventually shared by them, deals with how this study contributes to place-based education pedagogy. It basically shows how the students have become active participants in their respective rice-farming communities by sharing what they learned about CC and its impacts on rice production with their farmer-parents or other farmers in their communities. In sum, it tackles how students, if properly mobilized, can act on something that directly concerns them or their respective communities.

### Teaching tools and/or strategies

This subsection discusses the tools/strategies found effective in teaching CSRA to high school students. The general observation is that different tools/strategies are useful in specific contexts. One-size-fits-all tools/strategies must be avoided.

**Fig. 2:**
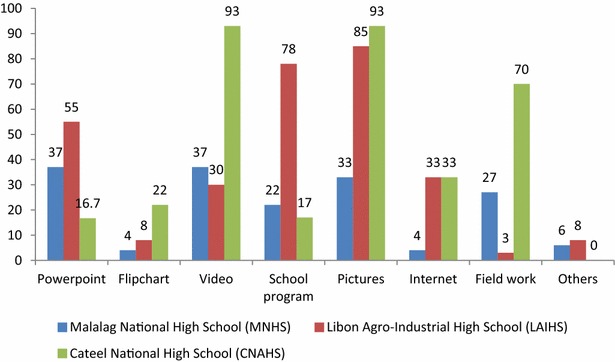
The teaching tools/strategies used in conveying climate-smart rice agriculture to students

Figure 2 offers several insights that must be seriously considered in bringing CSRA to students. First, use of visuals such as video (average of 53 %) and pictures (average of 70 %) is evident. FGDs with teachers note that videos are effective because they educate and entertain at the same time. The videos used the edutainment (education and entertainment) approach in discussing several climate-smart technologies and were in the Filipino language. These findings are similar to those reported by McNaught et al. (2014) in their Asia–Pacific study on how to best communicate CC. This result conveys the need for more developmental videos. On the other hand, while it is true that developmental videos are indeed effective in conveying CSRA messages, one must carefully consider the areas where they will be recommended, for instance, areas with no electricity. It is not safe to assume that most people have access to electricity. In some other Infomediary Campaign sites, electricity remains a luxury; hence watching videos may, at some point, put too much pressure on local resources. The bottom line is to be critical in assessing which of these methods can work in a given community.

Second, fieldwork was used extensively in CNAHS as reported by 70 % of its students. Its rice garden sits inside the school, with access to irrigation, which is favorable for rice farming. Based on interviews with the students, this seems to be an effective medium in conveying CSRA technologies. The literature strongly supports that young people learn best if they are presented with and are allowed to deal with real-world examples (UK STC 2007).

While student involvement is likely to result in favorable outcomes as far as CSRA integration is concerned, teachers must be reminded that timing is also crucial. Manalo et al. (2016) noted that planting rice when the sun is high is frowned upon by the students. Hence, this is something to which curriculum developers must pay close attention.

Third, pictures were the most preferred (average of 70 %) teaching tools. Teachers received instructional posters on harmful and beneficial organisms that they posted on the walls of their classrooms. These are highly visual instructional posters on the insects in Philippine ricefields. If compared with PowerPoint presentations, pictures can be used outright without need for any equipment. In the three schools for this study, LCD projectors were limited.

Table 1 shows the various methods and/or communication materials used in teaching CSRA in the three schools. The advantages and disadvantages in using each communication material are presented.

**Table 1:** Advantages and disadvantages of using different communication materials in teaching CSRA among high school students

Communication material	Advantages	Disadvantages
PowerPoint	Can contain plenty of information; highly visual, which is good for retention; ready to teach material (useful for busy teachers)	Requires equipment and electricity; issues on mastery, especially from the downloaded PowerPoint presentations
Flipchart (on tarpaulin)	Handy; can be used anywhere; highly visual; good for retention	Can be expensive to produce; contains limited information
Video	Handy; audiovisual; good for retention; entertainment value is high; shareable to android phones and tablets, which will work for tech-savvy students	Requires electricity and equipment; outdoor setup (using a projector in a wide place during daylight) can diminish quality and retention
School program	Effective in driving home the key messages of the advocacy; retention; maximizes student involvement; trains students to become good public speakers; leadership skills of the students can be honed if they are given certain tasks to perform; opportunity for community engagement is high; increase awareness among students who are not directly involved in the campaign; create linkages and collaboration with the local government unit and other partners	Requires a committed group of people willing to do many tasks; can be costly; resource-intensive; massive coordination needed
Photos/posters	Highly visual; facilitates retention; does not need electricity or equipment; can be referred to anytime the students need information	Lifespan variable; might be replaced every so often depending on how they are taken care of; limited information in one poster/photo
Internet	Contains massive information; updated materials	Internet connectivity is an issue in most rural communities; needs electricity and equipment; cost issues in access; information overload; information credibility is not always guaranteed
Fieldwork	Effective in actively engaging the students; increases confidence of the students to talk about rice-related technologies; can be an avenue to promote rice farming as something that is fun and engaging; high retention because of experiential learning	Wrong timing can lead to students not enjoying the activity; some costs involved (snacks and personal protective equipment of students and teachers); lack of farm equipment; unavailability of area for fieldwork

School programs were not very popular. But schools that had a unique and big program relating to CSRA encouraged good recall by the students of the technologies, such as in the case of LAIHS. School programs can be very engaging for the students, especially if they are given specific tasks to perform. At LAIHS, a farmers’ field day was conducted in which the school invited local executives, and the students performed various tasks. A field day is an occasion for showcasing rice production technologies to farmers in the community. This finding highlights that strong student involvement is key in CSRA integration. UK STC (2014) noted that impacts on development initiatives engaging the youth are far more pronounced if they are actively involved as opposed to being passive receivers of information.

We need to clearly evaluate the advantages and disadvantages of these different tools and/or strategies as applied in the local setting. While effectiveness must be a dominant criterion, several practical considerations must also be meticulously considered. For instance, video is by far very effective owing to its being an audiovisual medium. However, one should consider the capacity of the teachers to produce it, if materials are not supplied. Additionally, if electricity is a problem in an area, one may need to look for an alternative, as using video will certainly put pressure on local resources. Student involvement must also be prioritized while ensuring that people involved are not spread too thinly in the process.

### Push factors in teaching CSRA

This subsection discusses the key elements for successful CSRA integration in secondary schools. The data are drawn from the FGDs with teachers and key school officials and from field observations during site visits. Push factors are grouped into two: school/community-based factors, and those that refer to the key characteristics of the teachers.

As for community- and school-based factors, CSRA can easily be integrated if the school is located in a predominantly agricultural community, for our purposes a rice-farming community. The three schools for this study all share this characteristic (Table 2), which somehow relates to the success of their CSRA integration. Manalo et al. (2015b) noted that efforts to engage young people in rice farming are far more successful in key agricultural areas as opposed to non-agricultural areas. NRC (2000, p. 61) notes “learners of all ages are more motivated when they can see the usefulness of what they are learning and when they can use that information to do something that has an impact on others.”. The surrounding agricultural community of the school has provided the impetus for the ease of CSRA integration.

**Table 2:** Key elements for successfully engaging young people on CSRA

School	Physical environment	School environment	Characteristics of the teacher (education, outstanding traits)	Innovations implemented	Areas for improvement
Malalag National High School	Agricultural community where rice and vegetables are grown; generally rainfed	Supportive key school officials; teacher trained was given agri-related teaching load; has an estimated 3500 square meter (m^2^) rice garden beside the school	Has an agriculture-related degree; industrious; good networking skills; facilitative; shows high interest in the subject matter; reputable	Infomediary corner; engaged five other schools on his own by giving them seeds and all learning materials collected during the training; rice garden managed by the students; school bought pocket wi-fi so students can surf PinoyRice; active student-texters to the PhilRice Text Center (PTC)	ICT infrastructure in the area is poor, which limits the students from accessing online materials; frequent reshuffling of key school officials (and sometimes teachers)
Cateel National Agricultural High School	Agricultural community with mostly hilly areas; rice is generally planted in the lowland areas; timber production is practiced	Support from key school officials is variable; internet connectivity is a challenge; teacher trained was given agri-related teaching load; has a 3-hectare ricefield and 1000 m^2^ is being used for rice garden; receptive parent-teacher association members; school is 10 km from the the commercial center	Very intelligent (topped post-test twice during the training of teachers); highly innovative; has an agriculture-related degree; facilitative; industrious	Infomediary corner; loaning scheme for publications; rice garden maintained by the students	Frequent reshuffling of key school officials (and sometimes teachers)
Libon Agro-Industrial High School	Agricultural community where rice and other crops are grown; generally irrigated	Supportive key school officials and colleagues; teacher trained was given agri-related teaching load	Very intelligent (one of the topnotchers in the post-test during the training of teachers); coordination skills are high; facilitative; good working relationship with colleagues; has an agriculture-related degree; industrious	Field day participated in by private companies, farmers, and local government officials; rice garden outside the school managed by the students; recognized active infomediary students during the school’s recognition day; time alloted for students to register for the PTC	Rice garden is quite far from the school, which might discourage students from frequently visiting it; frequent reshuffling of key school officials (and sometimes teachers)

The second consideration that falls under the community- and school-based factors is the level of support extended by key school officials. This is not difficult to understand, as school-based activities cannot proceed without a good push from them. For instance, the teachers who underwent the CSRA training must be given a crop production load so they can integrate the learnings from the training. However, this is something that does not always happen. In past training programs under the Infomediary Campaign, some teachers were not given a crop production teaching load in the succeeding academic year, making it impossible for them to integrate the lessons from the training (Manalo et al. 2015a). Another case relates to some petty issues, which was noted in one of the three schools in this study. The teacher had difficulty engaging more students to participate, as the school head was not fully supportive.

All said, the school-based factors are actor-dependent. We have earlier stressed that the success of CSRA integration rests heavily on the approval and support of key school officials. While it is recognized in the literature that a supportive organization is central to the success of any development initiative (Bessette 2004), we find this problematic, as in the Philippines, school principals get reshuffled every so often. The continuity of programs and projects of the school usually suffers. The teachers, too, transfer from one school to another. Obviously, the expertise goes with the teacher trained. While re-echoing the lessons during the training is encouraged among the teachers, it does not always happen. Retraining new people has been a culture. Learning is sometimes retained by the trained teacher.

Teachers are a force to be reckoned with when it comes to CSRA integration. They are the ones who will teach the lessons and therefore serve as the key vehicle for the CSRA technologies to reach the students and eventually the farmers in the community. Perhaps this is why a number of studies on CC integration in the curriculum have looked into different areas concerning teachers such as their knowledge, perception, and attitudes toward CC (Lambert and Bleicher 2013; Ratinen et al. 2013; Liu et al. 2015). In this study, we have identified three push factors that are internal to the teachers—relevant education, strong interest, and innovativeness—drawn from our interviews and field visits.

The specialization of the teachers is important, as they are expected to exhibit mastery and competence when they talk of CSRA matters in their classrooms. All three teachers in this study have agriculture-related degrees (Table 2). Likewise, it might be difficult for non-agriculture teachers to catch up on some technical topics during the training, which would ultimately sacrifice the quality of training they can give to their students. Having some mastery of CSRA lessons will make the teachers effective CSRA communicators. Diehl et al. (2015) note that the best communicators must be mobilized to talk about CC **owing to the complexity of issues attached to it** (our emphasis).

Next to education is strong interest. The teachers must show keen interest in the subject matter before they can be effective. The three teachers trained did show this in various instances: they were among the top scorers during the post-test in the training on CSRA; they asked highly relevant questions during the training; they engaged their coteachers to support them in implementing the learnings from the training; and many others. The teachers, however, must keep themselves in check, as there had been cases in which teachers who were heavily passionate about CC tended to inject their own beliefs into their teaching (Liu et al. 2015). Some even looked for references to support their claims (Disinger 2001; Choi et al. 2010).

Last, innovativeness seems to be a hallmark of a champion CSRA teacher. Innovations refer to things the teachers did to better convey CSRA messages. The three schools under study did show plenty of innovations. An example is the MNHS teacher giving away seeds and learning materials to teachers in neighboring schools. All three schools also put up an Infomediary Corner in their respective libraries, containing materials on CSRA for easy access by the students. At CNAHS, the teacher even set up a loaning scheme so the students could bring home the knowledge products for their parents to read. This worked particularly well in instances when the concepts are just too complex for the students to understand. At LAIHS, the teacher initiated the holding of a videoconference through SKYPE so the students and the farmers whom they invited into their school (based in Albay Province in Southern Luzon) could consult with PhilRice experts in Nueva Ecija Province (Central Luzon). These innovations can be arrived at only by people who care to think about improvements. While innovations are highly encouraged, the teachers must ensure that they do not overdo it so it does not eat up their time for other school chores. It has been reported that inadequacy of time is among the major reasons for reluctance in integrating CC lessons in the curriculum (White et al. 2014).

While the key characteristics cited (relevant education, high interest, and innovativeness) are important, these must go with providing the best training opportunities for the teachers. Given that they are regarded as central to the success of CC integration, they must be trained technically so they become the best communicators of CSRA. Access to extension (Gbetibouo 2009; Di Falco et al. 2011; Truelove et al. 2015), information (Dang et al. 2014), and good relationship between the information sources and the recipients are positively linked to adaptation (IPCC 2007).

### Exogenous factors that may affect CSRA integration

While schools have all the means to influence their students, exogenous entities are equally powerful in shaping knowledge about CC. Such entities here refer to mass media and other institutions, private or public, which are also in the business of propagating knowledge on CSRA. Herman et al. (2015) noted that the mass media serve as the go-to source of information on CC. This is something that has been validated in our findings.

**Fig. 3:**
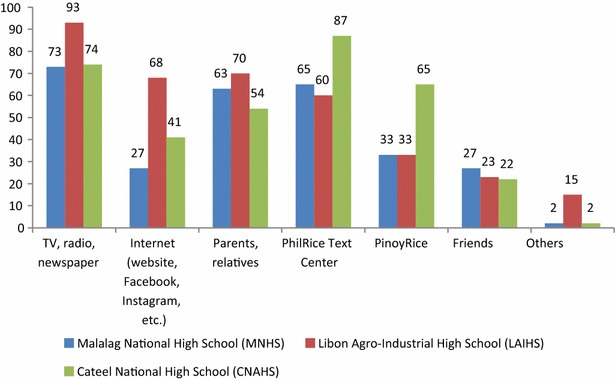
Sources of information on climate change by students

Figure 3 shows that in MNHS and LAIHS the traditional media (newspapers, radio, television) were the primary sources of CSRA information. Hence, initiatives to engage the schools must consider reinforcing efforts by ensuring that the mass media outlets are closely engaged. In the Philippines, where mass media command extensive power and clout over their audiences, careful and thorough engagement is necessary to ensure that the messages conveyed are based on science, and not on the platform of fear, which the mass media is prone to perpetuating.

In the same figure, students of CNAHS preferred the PTC as the source of information on CC, probably owing to the access to information the PTC facilitates. In the information and communications technology for development literature, among the reasons why people invest in mobile phones are speed of transaction and the significant reduction in costs associated with accessing information. This result is encouraging as far as information provision and access are concerned. In a country with a 103 cellular mobile telephone service density per 100 population in 2013 (PSA 2015), it pays to realize that this technology is being employed productively for development efforts. Additionally, this will afford the students higher quality information in terms of technical content, as the PTC has rice specialist agents who answer questions received by the facility. Interactions with the PTC agents can enhance learning. A minor criticism of the PTC is that it entails cost, but Manalo (2011) noted that young people’s spending for mobile phone prepaid credits in the Philippines was almost equal to their food expenditure. Given the unlimited texting offers in the Philippines, which makes mobile phone usage cheaper, young people will likely optimize this platform if they find it useful.

Another observation from Fig. 3 is that online sources have gained following. While this is a positive development, it should be noted that internet penetration in the Philippines remains low at 47 %, mostly in urban areas, where rice is not grown. Fast-tracking developments in information and communications technology (ICT) in the rural areas will lead to positive impacts on the information-seeking behavior of rural people, particularly the youth. Additionally, it should be highlighted that the research sites are far from town centers, where computer shops (telecenters) abound. On average, they spend about one US dollar just to access the internet. This is already a significant amount in the Philippines, where the rice-farming households remain poor (NSCB 2014). Hence, the key point for this finding is that, while it is good that online sources of information on CSRA have managed to gain some following, it should be matched by necessary improvements in ICT infrastructure if this is to fully take off on a large scale.

A key lesson from this subsection is that school engagement in CSRA cannot ignore external influences and drivers. These exogenous factors can reinforce CSRA messaging, and hence enhance learning, depending on how one utilizes them.

Having reflected on the tools/strategies, push factors, and exogenous factors that may affect integrating CSRA in the school curriculum, this research also looked at the types of information that are likely to be shared by students. Beyond integrating CSRA, the intended outcome is to see how young people can serve as information providers on CSRA in their respective rice-farming communities. The lessons taught in school must find their way into their communities to enhance the adaptive capacities of farmers. Hence, the next part scrutinizes the types of information that are likely to be shared by the students.

### Taught and shared

During the 2014–2015 academic year, 88 % of the students engaged from the three schools in this study performed as CSRA infomediaries. They either read, surfed, or texted information on CSRA and shared what they found with their farmer-parents or other farmers in their communities.

The PTC has student-texters from MNHS (400), CNAHS (305), and LAIHS (321). The numbers indicate that, aside from those actively engaged, other students have also been serving as infomediaries in their own capacities. The texters are grouped according to province; hence, the data below might be shared by other texters from other schools in the same province. Nonetheless, the figure seeks to convey that there are active texters from the schools covered as proof of their being infomediaries.

**Fig. 4:**
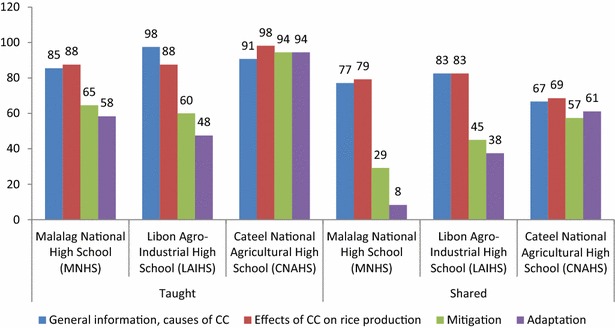
CSRA information taught and shared

This part looks at the dynamism of information transfer. It is important to look at how the students handled the information on CSRA that they received. General information on CC as well as on the effects of CC on rice production rated higher in terms of how they were perceived and shared by the students (Fig. 4). This can be explained by the level of complexity of the information contained in these categories. The higher-rating CSRA information was general knowledge in nature as opposed to the more technical mitigation- and adaptation-related information that talks about some how-to’s, which require some level of understanding about rice-farming operations that the student may not always possess.

The main implication of these findings is on message segmentation. It can be very tempting to teach everything to the students and expect them to go home ready to share the information with their farmer-parents or any farmer in their community. These findings put some limitation on what this initiative can competently do. It would do better to expect that a certain level of success can be expected if the more general sets of information will be passed on to the students. The more complex information can perhaps be better handled by their teachers and passed on to the farmers in the community using other media such as a farmers’ forum or other forms of information dissemination activities.

Alternatively, this is where reinforcement mechanisms become extremely useful. Other information outlets must be in place, such as the local media, the school itself holding community events, or the students tasked to promote the PTC among their parents and other farmers in the community. This way, students will not be compelled to understand highly complex topics on CSRA. Further, communicators might also need to look at how the information on the adaptation and mitigation modules is packaged.

The second main implication is on the level of emphasis that the teachers should give to various topics. Topics that are explained more in detail will certainly have a higher chance of being understood. Perhaps the teachers can spend more time discussing adaptation and mitigation mechanisms, and step up teaching by showing more examples and practical exercises. A word of caution on emphasis is for the teachers to be careful not to inject their own beliefs about CC, as has been observed in a study in the United States (Duschl 1990). The highest level of objectivity must be in place when discussing this matter to avoid misinformation.

**Fig. 5:**
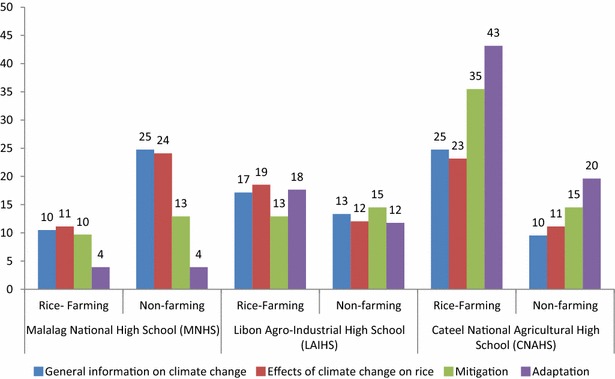
Disaggregated data on the information shared by students clustered according to household type (i.e., farming, nonfarming)

Breaking down the data will give a slightly different picture. Figure 5 shows the types of information the students shared relative to the source of livelihood of their respective households (i.e., farming or nonfarming). As it stands, 46 % of all the information types shared by CNAHS students were on adaptation and mitigation, 70 % of which was shared by students coming from rice-farming households. Hence, a revelation of these data is that such students can be relied on to share even complex information. This is consistent with earlier findings that students from rice-farming households, especially those who are top-performing in their respective classes, were able to explain complex rice-farming topics like pest dynamics to their parents and even to convince them to try certain technologies (Manalo et al. 2016). The source of livelihood will also have some bearing on the capacity of students to share far more complex information. Of the three schools, CNAHS had the highest percentage of students (65 %) coming from rice-farming households; (MNHS—32 %; LAIHS—47 %). Amartya Sen’s Capability Approach may be able to explain this, where it says that people will value something that they have a reason to value (Grunfeld 2007). The fact that these students came from families whose main livelihood source is rice farming indicates that they must have seen these CSRA modules as very useful for them. Hence, they must have exerted some effort to understand them so they could share the same with their parents.

## Conclusion

What this research has done is to unpack several issues in CSRA integration in the school curriculum, which is not well tackled in the literature. Several details to which practitioners must pay close attention are revealed. This area—how to teach CSRA or CC adaptation in general—offers fertile ground for research exploration owing to its urgency and complexity. What is cogent at this point is that there is wisdom in engaging teachers and students in efforts to disseminate CSRA information to farmers as evidenced by the sharing that transpired. This very same strategy can be used in tackling knowledge relating to CC adaptation.

Future research studies may consider a more thorough evaluation of teaching methods that can be used, message-framing and its effects on CC information dissemination in general, and a high-level risk communication discourse specific for teachers.
